# Sequential Portal Vein Embolization and Percutaneous Radiofrequency Ablation for Future Liver Remnant Growth: A Minimally Invasive Alternative to ALPPS Stage-1 in Treatment of Hepatocellular Carcinoma

**DOI:** 10.3389/fsurg.2021.741352

**Published:** 2021-09-30

**Authors:** Qiang Wang, Yujun Ji, Torkel B. Brismar, Shu Chen, Changfeng Li, Jiayun Jiang, Wei Mu, Leida Zhang, Ernesto Sparrelid, Kuansheng Ma

**Affiliations:** ^1^Division of Medical Imaging and Technology, Department of Clinical Science, Intervention and Technology (CLINTEC), Karolinska Institutet, Stockholm, Sweden; ^2^Division of Radiology, Department of Clinical Science, Intervention and Technology (CLINTEC), Karolinska Institutet, Karolinska University Hospital, Stockholm, Sweden; ^3^Institute of Hepatobiliary Surgery, Southwest Hospital, Army Medical University, Chongqing, China; ^4^Department of Radiology, Southwest Hospital, Army Medical University, Chongqing, China; ^5^Division of Surgery, Department of Clinical Science, Intervention and Technology (CLINTEC), Karolinska Institutet, Karolinska University Hospital, Stockholm, Sweden

**Keywords:** portal vein embolization (PVE), radiofrequency ablation (RFA), hepatocellular carcinoma (HCC), future liver remnant (FLR), liver resection (LR)

## Abstract

**Background:** To evaluate the feasibility and efficacy of sequential portal vein embolization (PVE) and radiofrequency ablation (RFA) (PVE+RFA) as a minimally invasive variant for associating liver partition and portal vein ligation for staged hepatectomy (ALPPS) stage-1 in treatment of cirrhosis-related hepatocellular carcinoma (HCC).

**Methods:** For HCC patients with insufficient FLR, right-sided PVE was first performed, followed by percutaneous RFA to the tumor as a means to trigger FLR growth. When the FLR reached a safe level (at least 40%) and the blood biochemistry tests were in good condition, the hepatectomy was performed. FLR dynamic changes and serum biochemical tests were evaluated. Postoperative complications, mortality, intraoperative data and long-term oncological outcome were also recorded.

**Results:** Seven patients underwent PVE+RFA for FLR growth between March 2016 and December 2019. The median baseline of FLR was 353 ml (28%), which increased to 539 (44%) ml after 8 (7–18) days of this strategy (*p* < 0.05). The increase of FLR ranged from 40% to 140% (median 47%). Five patients completed hepatectomy. The median interval between PVE+RFA and hepatectomy was 19 (15–27) days. No major morbidity ≥ III of Clavien-Dindo classification or in-hospital mortality occurred. One patient who did not proceed to surgery died within 90 days after discharge. After a median follow-up of 18 (range 3–50) months, five patients were alive.

**Conclusion:** Sequential PVE+RFA is a feasible and effective strategy for FLR growth prior to extended hepatectomy and may provide a minimally invasive alternative for ALPPS stage-1 for treatment of patients with cirrhosis-related HCC.

## Introduction

Hepatocellular carcinoma (HCC) ranks as the sixth most common cancer and the fourth cause of cancer-related death worldwide (1). Liver resection remains a potential curative treatment for HCC (2, 3). However, due to the latent nature of HCC, many patients may have developed an advanced disease when first diagnosed. For these patients, one of the main limiting factors for liver resection is the future liver remnant (FLR). Insufficient FLR increases the risk of posthepatectomy liver failure (PHLF), which is a main cause of perioperative mortality. In the normal liver, the minimum of FLR is 25–30%, while in cases of liver cirrhosis, the FLR should be at least 40% to ensure a safe liver resection (4).

To increase the FLR volume and expand the candidates of HCC patients for liver resection, many strategies have been proposed to induce FLR growth, such as portal vein embolization (PVE) or ligation (PVL). Among them, PVE has been regarded as a standard procedure prior to extended liver resection. Unfortunately, PVE results in a slow hypertrophy of the FLR, where a growth of 42–49% can be observed after 4–6 weeks (5). During this waiting period the tumor may progress and metastasize.

In recent years, associating liver partition and portal vein ligation for staged hepatectomy (ALPPS) emerged as a novel strategy for FLR growth before extended liver resection (6). During ALPPS stage-1, one branch of portal vein is ligated (mainly the right one), followed by liver parenchyma transection. This additional procedure for liver partition creates an amazing consequence: it promotes an FLR increase of 47–100% within 6–16 days (7). This interesting clinical phenomenon has sparked enthusiasm and extensive discussion in the hepatobiliary community, but also brings in criticism as its morbidity and mortality are fairly high (8). In the initial reports, the morbidity and mortality were as high as 68 and 12% respectively (9).

To reduce the incidence of perioperative complications and minimize the surgical trauma, plenty of technical variants for classic ALPPS have been proposed. For instance, a technique to use radiofrequency/microwave ablation or tourniquet to virtually split the liver parenchyma during ALPPS stage-1 has been developed and shown to have similar FLR growth as the ALPPS technique (10–12). Other technical variants include hybrid-ALPPS, TIPE-ALPPS and mini-ALPPS, which combine PVE and surgical partition of liver parenchyma for the FLR growth (13). However, these modified techniques still involve significant surgical trauma as they require two laparotomies. This may interfere with the physiologic condition of HCC patients significantly as these patients are often accompanied by cirrhosis with a marginally decompensated liver function. Development of a minimally invasive variant of ALPPS stage-1 that maintains the strengths of ALPPS for rapid liver growth would be of great value for the treatment of HCC patients.

Radiofrequency ablation (RFA) is a minimally invasive, safe and repeatable procedure, commonly used in patients with small HCC. We here propose a strategy to use PVE to substitute for PVL in classic ALPPS stage-1 and sequentially adopt percutaneous RFA for the liver tumor. We assumed that the additional RFA may trigger an inflammatory response, similar to that observed after surgical or virtual liver partition in classic or modified ALPPS stage-1. Such a strategy of sequential PVE+RFA can theoretically reduce the procedure-related morbidity and mortality while maintaining the advantages of classic ALPPS for rapid FLR growth. Therefore, the main aim of this study was to evaluate the feasibility and efficacy of sequential PVE + RFA strategy in induction of FLR growth in treatment of patients with cirrhosis related HCC. Secondly, the procedure related morbidity, mortality and long-term oncological outcome was assessed.

## Materials and Methods

### Study Design

This retrospective study was approved by the Ethics Committee of Southwest Hospital Chongqing, China (No. KY2020178). Due to the property of a retrospective study, no informed consent was waived. Clinical data was retrospectively collected from the Hospital Information System.

### Study Subjects

Clinically-diagnosed HCC patients with insufficient FLR (<40%) who underwent sequential PVE + RFA for the purpose of FLR hypertrophy prior to liver resection were enrolled in this study. The patients were in otherwise good condition with no evidence of extrahepatic metastasis during preoperative workup. Patients who had transcatheter arterial chemoembolization were excluded in this study.

### Liver Volumetry

Liver volumetry was obtained on the contrast-enhanced computed tomography (CT) imaging. The baseline of total liver volume and FLR volume, as well as their dynamic changes after the procedures, were determined by one researcher with 10 years of experience of abdominal imaging evaluation. The standardized total liver volume (sTLV) was calculated according to the following formula (14):


$$\begin{array}{l} \text{sTLV}=706.2\;*\text{body surface area }\left({\text{m}}^{2}\right)+\;2.4 \end{array}$$

The standardized FLR was defined as: FLR volume/sTLV ^*^100%. The FLR volume change was determined by: (FLR Vol_after_ – FLR Vol_0_)/FLR Vol_0_^*^100%, where FLR Vol_0_ stands for baseline FLR volume and FLR Vol_after_ the FLR volume after any procedures. It was regarded as a safe level to proceed to the liver resection when the FLR reached at least 40%.

### PVE Procedure

For all patients, percutaneous PVE was carried out via a contralateral transhepatic approach routinely. Under local anesthesia, the left branch of the portal vein was punctured under ultrasound (US) guidance. A 5F catheter sheath (RS^*^A50K10SQ Radifocus Introducer II; Terumo, Japan) together with a 5F Cobra catheter (RF^*^DB5508M, Terumo, Japan) was introduced in the portal vein with the end of the latter in the superior mesenteric vein to allow a portography. The embolization was performed using a combination of PVA microparticles (Cook Medical, Bloomington, IN) and coils (MWCE-35-14/10/6-NESTER, Nester Embolization Coil, Cook Medical, the U.S). Embolization of each right portal vein branch was carried out using antegrade method until the right portal system was occluded completely. The effect of PVE was checked by portography at the end of the operation. Absorbable gelatin sponge was used to prevent bleeding through the puncture tract when drawing back the catheter sheath and the catheter.

### RFA Procedure

**Figure 1:**
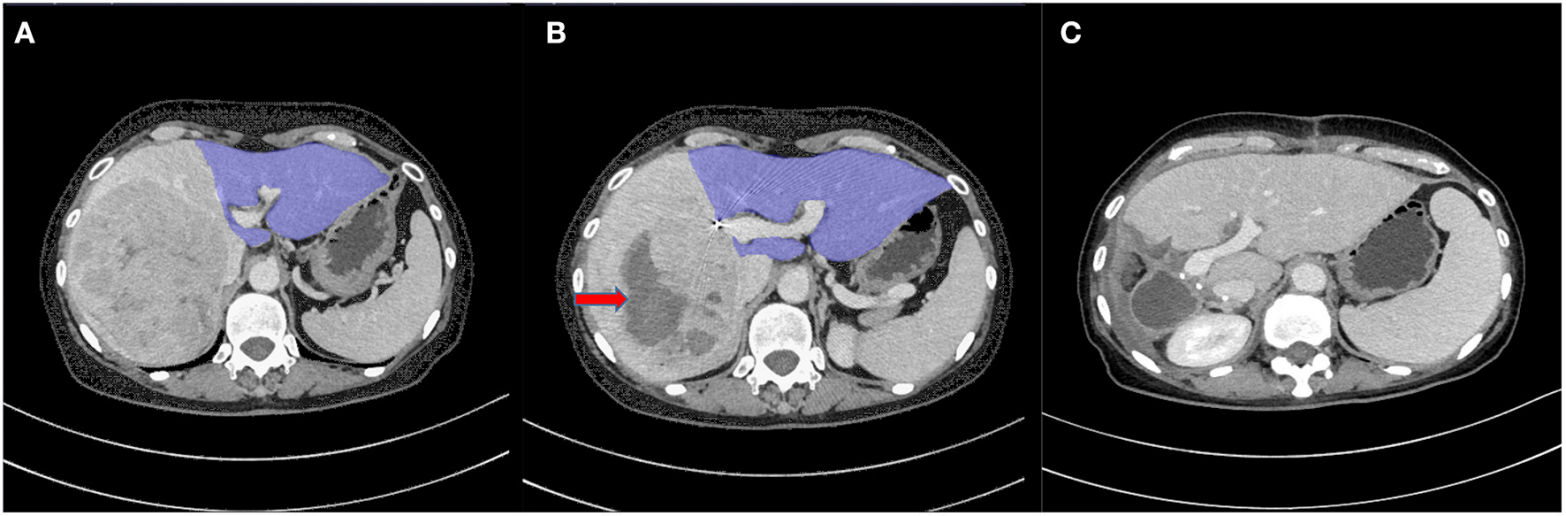
**(A)** Contrast-enhanced CT image before sequential portal vein embolization (PVE) + radiofrequency ablation (RFA) (PVE+RFA) shows a 106 mm × 103 mm tumor in the right lobe. The future liver remnant (FLR) (the blue part) was 385 ml (32%). **(B)** Eight days after PVE+RFA, the FLR had increased to 508 ml (42%) (the blue part). Red arrow shows tumor necrosis induced by RFA. **(C)** CT image at follow-up 48 days after liver resection shows a continued FLR growth.

Percutaneous RFA was carried out roughly within 3 days after PVE. When performing RFA, the patient was first evaluated by contrast-enhanced ultrasound (CE-US) to determine the location and size of the tumors, as well as the puncture site. Under conscious sedation and local anesthesia, two radiofrequency electrodes (LDDJS3-0200300A, Lide Co. Mianyang, Sichuan, China) were placed into the tumor with in-between tip distance of around 2 cm with the help of US guidance. After each ablation session, the electrodes were withdrawn a little for the next session of ablation. For each ablation session, the time was 6 min, with 3 sessions (18 min) in total. The whole process of liver tissue ablation was monitored by real-time US. When drawing out, the electrodes were heated to coagulate the tissue with an aim to stop bleeding and prevent possible track metastasis (Figure 1).

### Liver Resection

After PVE and RFA, the FLR volume was evaluated roughly every week through contrast-enhanced CT. When the FLR reached a safe level and the general condition of the patients was clinically stable, the tumor resection was performed. The patients were in the supine position and an inverted L-shaped incision was made in the right upper quadrant under general anesthesia. After abdominal cavity exploration, an intraoperative ultrasound exam was conducted to evaluate actual tumor margin. After cholecystectomy had been carried out, the right hepatic pedicle was separated, followed by ligation of the right branch of the portal vein. Intraoperative RFA (Habib 4X, RITA Medical Systems, Inc. CA, the US) was carried out between the FLR and the deportalized liver lobes.

Using a clamp-crushing technique, liver parenchyma was divided between the FLR and the deportalized lobes along the avascular area created by RFA. Liver partition was reached until the front wall of inferior vena cava. Then an endo-GIA linear vascular stapler was adopted to cut and suture the right hepatic pedicle. The deportalized liver lobes were dissected after the right hepatic ligament was detached. The abdominal wall was then closed after placing two silicone tubes for drainage.

### Perioperative Morbidity, Mortality and Follow-Up

Postoperative complications were evaluated using the Clavein-Dindo classification system and Grades III, IV and V were defined as major complications (15). Posthepatectomy liver failure (PHLF) was diagnosed according to “50–50” criteria, in which PHLF occurs when prothrombin time is less than 50% and serum bilirubin more than 50 μmol/L on postoperative day 5 or later (16). All deaths during hospitalization were taken into account as mortality, no matter related to the procedures or not.

When discharged, the patients were followed up by the Clinical Research Center monthly during the first 3 months and every 3 months thereafter.

### Statistical Analysis

Continuous variables were expressed as median with range, while categorical variables were presented as percentage. Nonparametric Wilcoxon signed-rank test was used to evaluate the FLR increment before and after PVE+RFA, with *p* < 0.05 regarded as a significant level. The software R (version 4.0.3, R Foundation for Statistical Computing, Vienna, Austria) was used to perform the statistical analysis.

## Results

Seven patients underwent sequential RFA +PVE for FLR growth prior to hepatectomy between March 2016 and December 2019. Of them, six patients were male and one female. The median age was 53 years (range 22–68). All patients had a history of HBV-related hepatitis and five had developed cirrhosis. Child-Pugh Grade A (5–6) scores was observed in all patients. The median rate of ICG-R15 was 3% (range 1–11%). Table 1 illustrates the detailed baseline information of the seven patients.

**Table 1:** Basic information of the patients.

**Patientno**.	**Sex**	**Age (years)**	**BMI (kg/m^**2**^)**	**Hepatitis (HBV-related)**	**Child-Pugh grade and score**	**MELDScore**	**ICG-R15 (%)**	**Fibrosis grade^**#**^**	**AFP (ng/mL)**
1	M	68	19.9	Yes	A(6)	9	4	6	2
2	M	58	19.4	Yes	A(5)	8	5	5	66
3	M	22	21.9	Yes	A(5)	6	1	4	57
4	M	48	20.9	Yes	A(5)	7	11	6	> 800
5	M	53	22.1	Yes	A(5)	7	1	6	> 800
6	F	61	20.2	Yes	A(5)	6	2	6	112
7	M	53	20.5	Yes	A(5)	6	3	6	37
Summary	6:1 (M/F)	53 (22–68)	20.5 (19.4–22.1)	–	–	7 (6–9)	3(1–11)	6 (4–6)	–

# *classified according to the Ishak scoring system; AFP: alpha fetoprotein; BMI, body mass index; HBV, hepatitis B virus; ICG-R15, indocyanine green test retention rate at 15 min; MELD, model for end-stage liver disease. The summary data is expressed as median with ranges*.

All patients were clinically diagnosed as HCC. Five patients had single tumor in the right lobe and two patients had multiple tumors. The median diameter of the tumors was 84 mm (range from 54 to 128 mm). One patient had a tumor thrombus in both the portal vein and the hepatic vein. No patient showed lymph nodes or extrahepatic metastasis in the admission workup (Table 2).

**Table 2:** Tumor characteristics.

**Patientno**.	**Location**	**Tumor number**	**Tumor size (mm)^§^**	**PVTT**	**HVTT**	**Pathology^**#**^**
1	Right lobe	1	78	No	No	Well differentiated HCC
2	Right lobe	1	128	No	No	HCC+Cholangiocarcinoma
3	Right lobe	Multiple	84	No	No	–
4	Right lobe	1	86	Rightanterior branch	Right hepatic vein	–
5	Right lobe	1	58	No	No	Moderately differentiatead HCC
6	Right lobe	1	106	No	No	Moderately-well differentiated HCC
7	Right lobe + Caudate lobe	Multiple	54	No	No	Moderately differentiated HCC

§ *refers to the maximum diameter of the tumor(s)*;

# *Two patients did not complete the hepatectomy; PVTT, portal vein tumor thrombus; HVTT, hepatic vein tumor thrombus; HCC, hepatocellular carcinoma*.

### Dynamic Volume Changes of FLR

**Figure 2:**
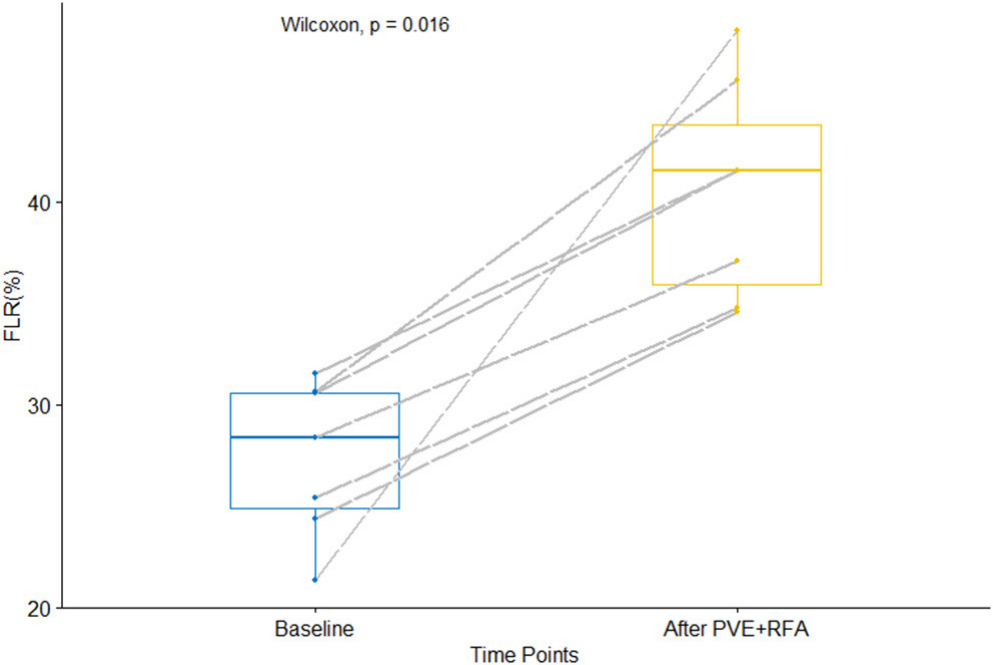
The volume of the future liver remnant in % of standardized total liver volume (sTLV), before and after sequential portal vein embolization (PVE) and radiofrequency ablation (RFA) (PVE+RFA) (*p* < 0.05).

The baseline FLR volume was 353 ml (258–399 ml), accounting for 28% (21–32%) of sTLV. A median of 8 days (range 7–18) after PVE+RFA, the FLR increased to a median of 508 ml (422–585 ml), with a corresponding FLR of 42% (35–48%, *p* < 0.05) (Figure 2). Prior to the liver surgery, the FLR volume of the five patients who proceeded to hepatectomy was 539 ml (507–620 ml) with a corresponding sTLV percentage of 44% (41–51%) (*p* = 0.06). The FLR increase ranged from 40 to 140% (median 47%) (Table 3).

**Table 3:** FLR changes before and after PVE+RFA.

	**Value**
	**(Median with range)**
Variable	
FLR, ml	353 (258–399)
FLR, %	28% (21–33%)
After PVE+RFA	
FLR, ml	508 (422–585)
FLR, %	42% (35–48%)
Before liver resection^#^	
FLR, ml	539 (507–620)
FLR, %	44% (41–51%)
Increase rate^#^	
FLR, ml	189 (154–362)
FLR, %	47% (40–140%)
Interval period, days	19 (15–27)
Completion rate	5/7

# *five patients completed hepatectomy; FLR, future liver remnant; PVE, portal vein embolization; RFA, radiofrequency ablation*.

The median interval between PVE and RFA was 3 days (ranging from 0 to 13 days). In two patients the procedure of RFA had to be postponed for 6 and 13 days after PVE due to low fever or abnormal liver function respectively. It is of note to point out that in these two patients, the initial FLR growth rate after PVE was low (31% and 8%, respectively), but then increased to 72 and 32% respectively after RFA was performed. The median interval from the PVE+RFA to hepatectomy was 19 days with a range from 15 to 27 days (Table 3).

### Complications and Mortality

No in-hospital death occurred. After the procedure of PVE+RFA, three patients experienced six adverse events with transient fever (less than 38.0°C) (*n* = 3), pleural effusion (*n* = 1), hypoalbuminemia (*n* = 1) and hyperbilirubinemia (*n* = 1). Among the five cases with hepatectomy, one patient developed pulmonary infection and one experienced abdominal infection; both recovered after conservative treatment (Clavien-Dindo classification < IIIb). No PHLF occurred after liver resection. The changes in liver function related indices during the perioperative period are demonstrated in Supplementary Table 1.

Two patients did not proceed to the hepatectomy: one patient experienced a persistently abnormal liver function (not reaching the definition of PHLF) and therefore received another palliative RFA. His FLR had increased to 42% at discharge. The other patient refused any further treatments after PVE+RFA, albeit the FLR had reached a safe level (46%).

### Intraoperative Data

Five patients eventually underwent liver resection (71%, 5/7). During the liver surgery, the median blood loss was 400 ml (300–600 ml), with 2 U (400 ml) of red blood cells transfused in one case. The median operative time was 310 min (290–360 min) (Table 4). Right hemihepatectomy was performed in four patients, extended hemihepatectomy in one patient. All patients underwent R0 resection with 1.0 cm negative margin. The pathologic exam for the resected lesion indicated that four patients had HCC and one had a mixture of HCC and cholangiocarcinoma (Table 2).

**Table 4:** PVE+RFA and liver resection related information.

**PatientsNo**.	**Time between PVE and RFA (days)**	**Time between PVE and CT scanning after RFA (days)**	**Time between PVE and hepatectomy (days)**	**Time betweenRFA and hepatectomy(days)**	**Blood loss(ml)**	**Operation time(min)**	**RBC tranfusion (ml)**
1	1	7	20	11	400	310	400
2	13	18	28	15	400	330	No
3	4	7	-	-	-	-	-
4	3	8	-	-	-	-	-
5	6	11	21	15	600	300	No
6	2	8	21	19	300	360	No
7	0	7	27	27	400	290	No
Summary	3 (0–13)	8(7–18)	21 (20–28)	15(11–27)	400 (300–600)	310(290–360)	–

*FLR, future liver remnant; PVE, portal vein embolization; RFA, radiofrequency ablation. The summary data is expressed as median with ranges*.

### Follow-Up and Oncological Outcome

The median overall survival (OS) for the seven patients was 18 months (range 3–50 months) as of the last follow-up in May 2020 and the survival rate was 71% (5/7). One patient who refused hepatectomy died within 90 days after discharge. The other patient developed multiple intrahepatic recurrence 11 months after liver resection and died 7 months later (18 months after liver resection). The median disease-free survival (DFS) was 12 months (range 3–50 months) after RFA procedure and 11 months (range 2–50 months) after liver resection.

## Discussion

The present study demonstrated that sequential PVE + RFA was a feasible and effective strategy to trigger FLR growth. Its efficacy for promoting FLR growth is comparable to that in classic ALPPS stage-1 in patients with HBV-related HCC (4, 17), despite being minimally invasive. To the best of our knowledge, this is the first report of using sequential PVE+RFA strategy for FLR growth before liver resection.

Up to date, the mechanism underlying ALPPS for introducing a rapid liver growth is not well established. The pathophysiologic alteration after ALPPS stage-1 includes the hemodynamic changes after portal vein occlusion and the regional surgical trauma during liver parenchyma transection, while the occluded deportalized liver serves as an auxiliary liver during the interval period (18). The rapid liver growth might be explained by an exclusive transport of inflammatory factors caused by the liver transection, along with various nutrients from the portal vein system, into the FLR through the non-occluded portal vein branch.

In fact, previous studies have illustrated the vital role of inflammatory factors in triggering liver growth. In an animal study, Schlegel et al. demonstrated that injection of plasma from ALPPS-treated mice into mice after PVL only created a same effect on FLR growth as after ALPPS. Unexpectedly, RFA on lung, spleen or kidney in mice after PVL can also produce a comparable FLR growth (19). This implies that certain cytokines or growth factors caused by surgical trauma or ablation could play a crucial role in liver regeneration after ALPPS. In the level of clinical practice, a study illustrated that partial transection of liver parenchyma (50–80%) in ALPPS stage-1 promoted comparable FLR growth as in classic ALPPS (100% liver transection) (20). That study further supports the inflammatory reaction is of importance for FLR growth in ALPPS, rather than liver parenchyma partition.

Based on these studies and assumptions, we postulated that using PVE to substitute for PVL along with percutaneous RFA for tumor to stimulate an inflammatory reaction may achieve the same effect of FLR growth as in classic ALPPS stage-1. The results of this study support this assumption, in which a median FLR increase of 47% was observed. This is comparable to other studies in HCC patient population (17, 21). It should be noted that percutaneous RFA in this strategy is not performed for the purpose of complete ablation of the tumors, therefore large tumors (>5 cm) or multiple tumors are also amenable for ablation.

The advantages of sequential PVE + RFA strategy in treatment of cirrhosis-related HCC with insufficient FLR are obvious: (1) two open, large and complex operations in classic ALPPS are limited to a single surgery, reducing both the surgical trauma and the patient's physical and psychological burden. (2) by avoiding the initial liver partition the corresponding complications between stage 1 and 2 are avoided. This is of great value, as initial research of ALPPS reported biliary leakage and resultant peritoneal infection caused by liver partition to be the main reasons for morbidity and mortality (18). In the present study no patients developed major complications. (3) as no manipulation on the liver is performed prior to hemihepatectomy, development of adhesions in the peritoneal cavity can be avoided; (4) with no manipulation on the tumor-bearing lobes the risk of tumor spread may decrease, (5) cauterization of the tumor can result in tumor necrosis. This will shrink the tumor size and might facilitate liver resection, especially in the case of huge liver tumor.

A similar strategy for the growth of insufficient FLR was proposed by Hong et al. (22), where percutaneous microwave ablation (MWA) and PVE were performed sequentially to minimize the trauma of classic ALPPS stage-1. They coined it as PALPP (percutaneous microwave ablation liver partition and portal vein embolization) (22). In that case report on a patient with hepatitis virus related HCC, PALPP yielded an FLR increase of 41% after 13 days. The same group later reported another PALPP case with an FLR increase of 50% (23). Those outcomes are similar to our results. The main difference between PALPP and sequential PVE + RFA is the ablated site: at PALPP the future resection plane is ablated while at PVE+RFA it is the tumor. Considering that they may have a similar effect for FLR growth, cauterization for the tumor seems more reasonable. Another practical issue of PALPP is that the technique of performing RFA to create an avascular area is difficult. The requirement of proficient surgical skills and the risk of middle hepatic vein damage may hamper a wide application of PALPP in clinical practice. Another research adopted a similar strategy as ours by using PVE first, followed by MWA for FLR growth, but using MWA for future resection plane (24). The median FLR growth rate in the included three cases was 45%, and no severe complications occurred (Table 5A). Regarding basic research, until now only two animal studies have investigated the strategy of PVE + RFA/MWA. One proved the feasibility of this approach in a porcine model (25) and the other in a rabbit model (26) (Table 5B). Future clinical and experimental research is urgently required to explore the underlying mechanism of the strategy of PVE+RFA.

**Table 5:** Summary of studies about the strategy combining PVE and RFA/MWA for FLR growth.

**A. CLINICAL RESEARCH**													
**Study ID**	**Year**	**Region**	**Research type**	**Cases**	**Strategy**	**Indication**	**Baseline FLR**	**FLR increase rate**	**Interval between the two procedures**	**Interval between intervention and hepatectomy (days)**	**Complications**	**Completion rate**	**In-hospital mortality**
Hong et al. (22)	2016	China	Case report	1	MWA+PVE (PALPP)	HCC	28.9%	41.2%	3	14	mild fever mild ascites	1/1(100%)	0
Liu et al. (23)	2017	China	Case report	1	MWA+PVE	Liver metastasis	34%	50%	N.A	10	N.A	1/1(100%)	0
Lunardi et al. (24)	2018	Italy	Case series	3	PVE+MWA (PISA)	2 Cholangiocarcinoma,1 Liver metastasis	24.6% (mean)	45.1%	21 days	21,12, N.A	0	2/3(67%)	0
**B. EXPERIMENTAL RESEARCH**													
**Study ID**			**Year**	**Region**	**Animal type**		**Animal number**	**Strategy**	**FLR**	**Complicates**		**Conclusion**	
Gimenes et al. (25)			2018	France	Pigs		4	PVE + RFA (PRALPPS)	N.A	No procedure-related complications; all survived		PRALPPS is a feasible technique	
Gaba et al. (26)			2017	USA	Rabbits		8 vs. 7 controls of PVE (after learning curve exclusion)	PVE+MWA	Dry weight: 0.32 g vs. 0.29 g (PVE+MWA vs. PVE, with *p* < 0.05)	One rabbit died in the learning-curve period		PVE+MWA is a feasible technique and may trigger a greater liver growth compared with PVE	

*FLR, future liver remnant, MWA, microwave ablation, N.A, not available, PVE, portal vein embolization, RFA, radiofrequency ablation*.

In recent years, another strategy with the same aims of PVE+RFA have also been proposed, such as PVE with hepatic vein embolization (HVE) (27, 28). Alternative names are biembolization or liver venous deprivation (LVD) (27, 28). It can be sequentially or concurrently performed. Although initial studies have shown that PVE+HVE is a safe and feasible strategy, HVE is a complex and delicate procedure which should be performed by experienced interventional surgeons. By contrast, percutaneous RFA is a simple, minimally invasive and repeatable procedure, widely-used in clinical practice. The FLR increment in the two strategies (PVE+HVE vs. PVE + percutaneous RFA) is comparable, where PVE+HVE has resulted in a growth ranging between 41 to 44% (29, 30), while we report a 47% growth after PVE+RFA. However, the studies are small and future studies are warranted to compare the morbidity and efficacy between PVE+ RFA and PVE+HVE.

In this case series, there were two patients who did not complete the liver resection. One patient refused to receive any treatment after PVE+RFA, despite the FLR had increased to a sufficient size. This patient died within 90-day after discharge. For the other patient, the FLR also increased to a safe level after a waiting time of 19 days, but the liver function was continuously in the abnormal range. It was therefore decided to abandon the curative hepatectomy strategy. The patient underwent palliative RFA and other palliative care. Out of expectations, he survived as of the last follow-up (43 months after discharge).

There are some limitations to be acknowledged in this study. To begin with, there was no control group, making a direct comparison of efficacy between PVE+RFA and PVE alone impossible. However, the FLR increase after PVE among cirrhosis patients has been reported to be between 24 and 38% during a median of 36 days (31), which is inferior to our results. Still, prospective, controlled trials are required to verify the efficacy of sequential PVE+RFA. Secondly, this study is also limited by the patient number. Future studies with larger sample size and diverse indications (e.g. early-stage HCC with normal liver parenchyma) are needed to confirm our successful treatment rate and the long-term oncological outcome. Thirdly, the effect of PVE+RFA on the liver tumor remains unclear. The same uncertainty can also be seen in classic ALPPS or its variants. Until now the long-term oncological outcome of classic ALPPS is not well-established. Thorough, well-designed basic research is required to explore the microenvironment of the deportalized lobes after ALPPS step-1 or PVE+RFA. Fourth, the order of the procedures in PALPP and PVE+RFA differs. Whether this order affects the oncologic outcome of the HCC patient, and which one should be performed first needs more studies. Lastly, we did not make a comparison between PVE+RFA and classic ALPPS, RFA-assisted ALPPS, or any of the other ALPPS variants involving PVE, such as mini-ALPPS, hybrid-ALPPS or TIPE-ALPPS.

## Conclusions

To sum up, sequential PVE + RFA seems to be a safe and efficient approach to induce growth of FLR in the treatment of cirrhosis-related HCC.

## Data Availability Statement

The original contributions presented in the study are included in the article/Supplementary Material, further inquiries can be directed to the corresponding author/s.

## Ethics Statement

The studies involving human participants were reviewed and approved the Ethics Committee of Southwest Hospital Chongqing, China (No. KY2020178). Written informed consent for participation was not required for this study in accordance with the national legislation and the institutional requirements.

## Author Contributions

QW and YJ: writing–original draft preparation. TB and ES: writing–review and editing. KM: conceptualization. CL and JJ: methodology. QW and SC: formal analysis and investigation. QW, TB, and KM: funding acquisition. WM and KM: surgical and intervention operations. TB, LZ, and KM: supervision. All authors contributed to the article and approved the submitted version.

## Funding

This work was funded by National Natural Science Foundation of China (Nos. 82073346 and 81672857), The Chongqing Technology Innovation and Application Development Project (No. cstc2019jscx-msxmX0230), Clinical Research Fund of the Army Medical University (No. SWH2017ZDCX4101), and Famous Teachers section of the Chongqing Talents Program (4246ZP112). QW receives a scholarship from China Scholarship Council (CSC, No. 201907930009).

## Conflict of Interest

The authors declare that the research was conducted in the absence of any commercial or financial relationships that could be construed as a potential conflict of interest.

## Publisher's Note

All claims expressed in this article are solely those of the authors and do not necessarily represent those of their affiliated organizations, or those of the publisher, the editors and the reviewers. Any product that may be evaluated in this article, or claim that may be made by its manufacturer, is not guaranteed or endorsed by the publisher.

## References

[1] Villanueva A. Hepatocellular Carcinoma. N Engl J Med. (2019) 380:1450–62. 10.1056/NEJMra1713263 30970190

[2] Hocquelet A Balageas P Laurent C Blanc JF Frulio N Salut C. Radiofrequency ablation versus surgical resection for hepatocellular carcinoma within the Milan criteria: a study of 281 Western patients. Int J Hyperthermia. (2015) 31:749–57. 10.3109/02656736.2015.1068382 26365503

[3] Tang T Feng X Yan J Xia F Li X Ma K. Predictive value of indocyanine green retention rate with respect to complications of radiofrequency ablation in 878 patients with hepatocellular carcinoma. Int J Hyperthermia. (2014) 30:402–7. 10.3109/02656736.2014.951404 25256893

[4] Kishi Y Abdalla EK Chun YS Zorzi D Madoff DC Wallace MJ. Three hundred and one consecutive extended right hepatectomies: evaluation of outcome based on systematic liver volumetry. Ann Surg. (2009) 250:540–8. 10.1097/SLA.0b013e3181b674df 19730239

[5] Michal K Sau M Tamara GM Long JR. A better route to ALPPS: minimally invasive vs open ALPPS. Surg Endosc. (2020) 34:2379–89 10.1007/s00464-020-07437-3 32274625 PMC7214383

[6] De Santibanes E Clavien PA. Playing Play-Doh to prevent postoperative liver failure: the “ALPPS” approach. Ann Surg. (2012) 255:415–7. 10.1097/SLA.0b013e318248577d 22330039

[7] Lau WY Lai EC. Modifications of ALPPS - from complex to more complex or from complex to less complex operations. Hepatobiliary Pancreat Dis Int. (2017) 16:346–52. 10.1016/S1499-3872(17)60034-1 28823363

[8] Lang H. ALPPS - Beneficial or detrimental? Surg Oncol. (2019) 33:249–53 10.1016/j.suronc.2019.10.013 31740102

[9] Schnitzbauer AA Lang SA Goessmann H Nadalin S Baumgart J Farkas SA. Right portal vein ligation combined with in situ splitting induces rapid left lateral liver lobe hypertrophy enabling 2-staged extended right hepatic resection in small-for-size settings. Ann Surg. (2012) 255:405–14. 10.1097/SLA.0b013e31824856f5 22330038

[10] Wang Q Yan J Feng X Chen G Xia F Li X. Safety and efficacy of radiofrequency-assisted ALPPS (RALPPS) in patients with cirrhosis-related hepatocellular carcinoma. Int J Hyperthermia. (2017) 33:846–52. 10.1080/02656736.2017.1303752 28540784

[11] Robles R Parrilla P Lopez-Conesa A Brusadin R De La Pena J Fuster M. Tourniquet modification of the associating liver partition and portal ligation for staged hepatectomy procedure. Br J Surg. (2014) 101:1129–34. 10.1002/bjs.9547 24947768

[12] Jiao LR Fajardo Puerta AB Gall TMH Sodergren MH Frampton AE. Rapid induction of liver regeneration for major hepatectomy (REBIRTH): a randomized controlled trial of portal vein embolisation versus ALPPS assisted with radiofrequency. J Surg Oncol. (2019) 11:302. 10.3390/cancers11030302 30836678 PMC6468856

[13] Baili E Tsilimigras DI Moris D Sahara K Pawlik TM. Technical modifications and outcomes after Associating Liver Partition and Portal Vein Ligation for Staged Hepatectomy (ALPPS) for primary liver malignancies: a systematic review. Surg Oncol. (2020) 33:70–80. 10.1016/j.suronc.2020.01.010 32561102

[14] Urata K Hashikura Y Ikegami T Terada M Kawasaki S. Standard liver volume in adults. Transplant Proc. (2000) 32:2093–4. 10.1016/S0041-1345(00)01583-9 11120082

[15] Clavien PA Barkun J De Oliveira ML Vauthey JN Dindo D Schulick RD. The Clavien-Dindo classification of surgical complications: five-year experience. Ann Surg. (2009) 250:187–96. 10.1097/SLA.0b013e3181b13ca2 19638912

[16] Balzan S Belghiti J Farges O Ogata S Sauvanet A Delefosse D. The “50-50 criteria” on postoperative day 5: an accurate predictor of liver failure and death after hepatectomy. Ann Surg. (2005) 242:824–8. 10.1097/01.sla.0000189131.90876.9e 16327492 PMC1409891

[17] Wang Z Peng Y Hu J Wang X Sun H Sun J. Associating liver partition and portal vein ligation for staged hepatectomy for unresectable hepatitis B virus-related hepatocellular carcinoma: a single center study of 45 patients. Ann Surg. (2020) 271:534–41. 10.1097/SLA.0000000000002942 29995681

[18] Alvarez FA Ardiles V Sanchez Claria R Pekolj J De Santibanes E. Associating liver partition and portal vein ligation for staged hepatectomy (ALPPS): tips and tricks. J Gastrointest Surg. (2013) 17:814–21. 10.1007/s11605-012-2092-2 23188224

[19] Schlegel A Lesurtel M Melloul E Limani P Tschuor C Graf R. ALPPS: from human to mice highlighting accelerated and novel mechanisms of liver regeneration. Ann Surg. (2014) 260:839–46. 10.1097/SLA.0000000000000949 25379855

[20] Petrowsky H Gyori G De Oliveira M Lesurtel M Clavien PA. Is partial-ALPPS safer than ALPPS? A single-center experience. Ann Surg. (2015) 261:e90–92. 10.1097/SLA.0000000000001087 25706390

[21] Chan A Zhang WY Chok K Dai J Ji R Kwan C. ALPPS versus portal vein embolization for hepatitis-related hepatocellular carcinoma: a changing paradigm in modulation of future liver remnant before major hepatectomy. Ann Surg. (2019) 273:957–65. 10.1097/SLA.0000000000003433 31305284

[22] Hong De F Zhang YB Peng SY Huang DS. percutaneous microwave ablation liver partition and portal vein embolization for rapid liver regeneration: a minimally invasive first step of alpps for hepatocellular carcinoma. Ann Surg. (2016) 264:e1–2. 10.1097/SLA.0000000000001707 26967629 PMC4902319

[23] Liu J Zhang C Hong D Shang M Yao W Chen Y. Percutaneous microwave ablation liver partition and portal vein embolization for planned hepatectomy due to large gastrointestinal stromal tumor metastases: a case report. Medicine (Baltimore). (2017) 96:e8271. 10.1097/MD.0000000000008271 29049221 PMC5662387

[24] Lunardi A Cervelli R Volterrani D Vitali S Lombardo C Lorenzoni G. Feasibility of percutaneous intrahepatic split by microwave ablation (PISA) after portal vein embolization for hypertrophy of future liver remnant: the radiological stage-1 ALPPS. Cardiovasc Intervent Radiol. (2018) 41:789–98. 10.1007/s00270-018-1882-7 29359240

[25] Gimenez ME Houghton EJ Davrieux CF Serra E Pessaux P Palermo M. Percutaneous radiofrequency assisted liver partition with portal vein embolization for staged hepatectomy (Pralpps). Arq Bras Cir Dig. (2018) 31:e1346. 10.1590/0102-672020180001e1346 29513807 PMC5863995

[26] Gaba RC Bui JT Emmadi R Lakhoo J. Ablative liver partition and portal vein embolization: proof-of-concept testing in a rabbit model. J Vasc Interv Radiol. (2017) 28:906–12 e901. 10.1016/j.jvir.2017.02.011 28292634

[27] Kambakamba P Hoti E Cremen S Braun F Becker T Linecker M. The evolution of surgery for colorectal liver metastases: A persistent challenge to improve survival. Surgery. (2021). 10.1016/j.surg.2021.06.033. [Epub ahead of print]. 34304889

[28] Kim D Cornman-Homonoff J Madoff DC. Preparing for liver surgery with “Alphabet Soup”: PVE, ALPPS, TAE-PVE, LVD and RL. Hepatobiliary Surg Nutr. (2020) 9:136–51. 10.21037/hbsn.2019.09.10 32355673 PMC7188547

[29] Guiu B Chevallier P Denys A Delhom E Pierredon-Foulongne MA Rouanet P. Simultaneous trans-hepatic portal and hepatic vein embolization before major hepatectomy: the liver venous deprivation technique. Eur Radiol. (2016) 26:4259–67. 10.1007/s00330-016-4291-9 27090112

[30] Hwang S Lee SG Ko GY Kim BS Sung KB Kim MH. Sequential preoperative ipsilateral hepatic vein embolization after portal vein embolization to induce further liver regeneration in patients with hepatobiliary malignancy. Ann Surg. (2009) 249:608–16. 10.1097/SLA.0b013e31819ecc5c 19300228

[31] Lopez-Lopez V Robles-Campos R Brusadin R Lopez-Conesa A De La Peña J Caballero A. ALPPS for hepatocarcinoma under cirrhosis: a feasible alternative to portal vein embolization. Ann Transl Med. (2019) 7:691. 10.21037/atm.2019.10.57 31930092 PMC6944538

