# Supplementary material for: Sequential Portal Vein Embolization and Percutaneous Radiofrequency Ablation for Future Liver Remnant Growth: A Minimally Invasive Alternative to ALPPS Stage-1 in Treatment of Hepatocellular Carcinoma
Source: Front Surg. 2021 Sep 30;8:741352. doi: 10.3389/fsurg.2021.741352 (PMC8515047; doi:10.3389/fsurg.2021.741352)
Supplement: Supplementary file 1 [file Table_1.docx]

Supplement table 1. Liver function and other indices changes during perioperative time

| Index | Baseline | After PVE | After RFA# | Before liver resection§ | Postoperative day 5§ | Normal range |
| --- | --- | --- | --- | --- | --- | --- |
| ALT | 44  (29-81) | 99  (30-589) | 209  (128-424) | 78  (32-256) | 63  (28-133) | 0-42 (IU/L) |
| AST | 54  (35-125) | 141  (42-643) | 345  (221-663) | 84  (39-390) | 36  (20-50) | 0-42 (IU/L) |
| TBIL | 14  (10-17) | 21  (17-32) | 28  (15-42) | 14  (10-20) | 21  (13-29) | 6-21(umol/L) |
| Alb | 41  (35-45) | 35  (32-40) | 35  (30-97) | 34  (30-38) | 34  (28-41) | 38-51(g/L) |
| Cr | 71  (46-98) | 72  (38-98) | 62  (51-97) | 63  (41-96) | 62  (34-102) | 54-133(umol/L) |
| PT | 12  (10-13) | 13  (10-18) | 14  (13-15) | 11  (5-13) | 12  (2-16) | 11-15 (s) |
| ICG-R15(%) | 3  (1-11) | - | - | 4  (0-11) | - | - |

Note: # six patients’ data as one patient underwent PVE and RFA on the same day; §five patients’ data; Alb, albumin; ALT, alanine aminotransferase; AST, aspartate aminotransferase; Cr, creatinine; ICG-R15, indocyanine green retention rate at 15 min; TBIL, total bilirubin; PVE, portal vein embolization; PT, prothrombin time; RFA, radiofrequency ablation.
